# The urinary levels of CTX-II, C2C, PYD, and Helix-II increased among adults with KBD: a cross-sectional study

**DOI:** 10.1186/s13018-019-1392-6

**Published:** 2019-10-21

**Authors:** Quan Quan Song, Li Yan Sun, Chun Hui Li, Yu Jiao Liu, Si Lu Cui, Yun Qi Liu, Yan Hong Cao, Jun Rui Pei, Yue Wang, Wei Lian, Zhe Jiao, Qing Deng, Jun Yu

**Affiliations:** 1 Institute for Kashin-Beck Disease Control and Prevention, Chinese Center for Disease Control and Prevention, Harbin Medical University, Harbin, 150081 Heilongjiang China; 2 Department of Human Resource, The Affiliated Zhongshan Hospital, Xiamen University, Xiamen, 361004 Fujian China; 3 Key Laboratory of Etiology and Epidemiology, National Health and Family Planning Commission, Harbin, 23618504 Heilongjiang China

**Keywords:** Urinary biomarkers, Osteoarthritis, Kashin-Beck disease

## Abstract

**Background:**

Kashin-Beck disease (KBD) is an endemic osteoarthropathy, and its pathogenesis is still not entirely clear. Pathologically, many KBD changes are similar to those of osteoarthritis (OA). Therefore, this study aimed to identify changes in the levels of potential urinary biomarkers for OA, including C-telopeptide of type II collagen (uCTX-II), type II collagen cleavage neoepitope (uC2C), pyridinoline (uPYD), and uHelix-II, among adults with KBD.

**Methods:**

Urinary samples of 83 external control (EC) subjects, 91 KBD patients, and 86 internal control (IC) subjects were tested by ELISA after the subjects completed a questionnaire and X-ray examination.

**Results:**

The medians of the four markers in the KBD group were higher than those in the EC group and those in the IC group. The medians in the grade II KBD group were higher than those in the grade I group but were not statistically significant (*P* = 0.301, *P* = 0.408, *P* = 0.204, and *P* = 0.898 for uCTX-II, uC2C, uPYD, and uHelix-II, respectively). The area under the curve (AUC) of uCTX-II (0.775) was higher than that of the others (0.672, 0.639, and 0.628 for uC2C, uPYD, and uHelix-II, respectively).

**Conclusion:**

The levels of uCTX-II, uC2C, uPYD, and uHelix-II were elevated in adults with KBD and showed an increasing trend as the severity of KBD increased. The prediction accuracy of uCTX-II was more useful than that of the others for assisting in the diagnosis of KBD.

## Background

Kashin-Beck disease (KBD) is an endemic osteoarthropathy [1], and its pathological characteristics are multiple symmetric degeneration or necrosis of articular cartilage and secondary osteoarthritis. It even leads to shortened limbs and disability in severe cases. KBD is a severe osteochondrosis (OC) with onset in childhood. It is well known that adults with KBD became sick in childhood that it is difficult to recover from the condition and that it gradually worsens and develops into secondary osteoarthritis (OA).

KBD endemic areas are mainly distributed in a narrow strip from northeast China to the Qinghai-Tibet Plateau but have also spread to far east Russia and North Korea. Although the disease has been controlled effectively, there were still over 530,000 KBD patients and 1.16 million high-risk residents in 13 provinces in China according to a national annual report on endemic diseases in 2017.

Although KBD has been studied for more than 160 years, the pathogenesis of KBD is still not entirely clear and needs further exploration. Pathologically, bone and cartilage destruction, matrix degradation, and other pathological changes caused by KBD are similar to those of osteoarthritis (OA), which has garnered widespread attention and been studied for a particularly long time. C-telopeptide of type II collagen (uCTX-II, with amino acid sequence ^1049^EKGPDP^1054^) [2], type II collagen cleavage neoepitope (uC2C, with amino acid sequence ^787^GPOGPQG^794^) [3], pyridinoline (uPYD) [4], and uHelix-II [5] are all cartilage collagen degradation products in urine that have been used in many OA studies, and they are also relatively useful biomarkers for cartilage changes.

Therefore, these OA biomarkers were introduced into our KBD studies to explore the roles they play in assisting diagnosis of KBD. In this study, the levels of uCTX-II, uC2C, uPYD, and uHelix-II were compared between healthy controls and adult KBD patients. At the same time, we tried to further understand the mechanism for the development of KBD using these four indicators.

## Methods

### Selection of study sites

This field study was carried out in September 2014, and the study sites were selected in the historical endemic and non-endemic areas of KBD in Jilin Province and Heilongjiang Province based on the China Health Ministry’s historical monitoring data. Some KBD endemic areas, including Dongxia Village in Qian’an County, Zhoujia Village in Qianguo County, Hanxia Village and Youhao Village in Jiaohe County, and Yushucha Village and Pubanshi Village in Huinan County, were selected in Jilin Province.

The non-KBD endemic areas, including Sanjing Village in Qian’an County and Fuqiang Village in Jiaohe County in Jilin Province and Heigang Village in Longjiang County, were selected in Heilongjiang Province.

The above selected areas were rural areas with similar economic conditions. Both of their climatic conditions and living habits were similar. The local residents fed on rice and corn, and their nutritional status was also similar.

### Study subjects and contents

First, the morning urine samples (middle urine) of the subjects were collected. A questionnaire was completed by residents aged 40 years or older living in the study sites listed above. The specific contents of the questionnaire included height, weight, living habits, past medical history, and current medical history. Next, technical staff took frontal X-rays of the right hand and bilateral knee joints and lateral X-rays of the bilateral ankles of all participants. After the questionnaire and radiological examinations, eligible individuals were selected. The subjects in the KBD group (KBD) and in the internal control group (IC) were recruited in KBD endemic areas; to compare bone metabolism in KBD epidemic areas and non-endemic areas, external controls (ECs) were recruited from non-KBD endemic areas based on X-rays and the inclusion criteria for the study subjects.

KBD patients were diagnosed according to the Diagnosis of Kashin-Beck Disease (WS/T 207-2010) [6]. Residents with any of the following would be excluded. First, the subject did not suffer from other bone or cartilage diseases or other diseases that are known to affect bone metabolism (such as Paget’s disease, osteoporosis, osteomalacia, cancer, bone hyperplasia, femoral head necrosis, metacarpal arthritis, etc.). Second, the subject had no history of traumatic knee disease. Third, the metabolism of the liver and of the kidneys of the subject was normal. Fourth, the subject was not overweight (body mass index ≤ 30). Fifth, the subject had not received any hormones or other medications that affect bone metabolism. The control subjects did not suffer from KBD or OA and met the criteria listed above.

### Detection methods

The X-rays were obtained by a portable high-frequency digital radiography (DR) medical diagnostic detector (Beijing Langsafe Imaging Technology Co., Ltd). The levels of uCTX-II, uC2C, uPYD, and uHelix-II were quantified by enzyme-linked immunosorbent assay (ELISA) in accordance with the manufacturer’s instructions (uCTX-II and uC2C ELISA kits were from Shanghai Shifeng Biological Technology Co., Ltd.; uPYD and uHelix-II ELISA kits were from Shanghai Yuanye Biological Technology Co., Ltd). The concentrations of the four urine markers were corrected by urinary creatinine levels. The intra-assay and inter-assay CVs of all biomarkers were less than 15%.

### Statistical methods

SPSS software 20.0 was used for statistical analyses. Ages were expressed as the mean ± standard deviation (SD). The one-way analysis of variance (ANOVA) and the chi-square test (*χ*^2^) were used to compare the age distributions and the sex composition of the three groups, respectively. The levels of uCTX-II, uC2C, uPYD, and uHelix-II in each group were expressed as medians and quartiles. Different levels among the groups were compared using the Kruskal-Wallis *H* test. *P* < 0.05 was considered statistically significant. To investigate the diagnostic ability of each biomarker to diagnose KBD, receiver operator characteristic (ROC) curves were employed to display the sensitivity, specificity, and area under the curve (AUC) for all subjects.

## Results

### Demographic characteristics of the subjects

A total of 1688 people were initially investigated in the field. According to our inclusion criteria and based on matching principles for age and sex, 260 people were ultimately selected for analysis: 83 external control (EC) subjects (mean age 58.96 ± 5.81 years, 33 males, and 50 females), 86 internal control (IC) subjects (mean age 59.58 ± 5.11 years, 36 males, and 50 females), and 91 KBD patients (mean age 60.38 ± 5.08 years, 37 males, and 54 females) were tested by ELISA. There were no significant differences among the three groups in terms of age (*F* = 1.558, *P* = 0.213) or sex (*χ*^2^ = 0.078, *P* = 0.962) (Table 1).

**Table 1:** Demographic characteristics of the three groups

Demographic	Non-KBD areas	KBD areas		*H/χ* ^2^	*P*
	EC	IC	KBD		
Case number (*n*)	83	86	91	NA	NA
Age (year)	58.96 ± 5.81	59.58 ± 5.11	60.38 ± 5.08	1.558^a^	0.213^a^
Sex, male/female (*n*)	33/50	36/50	37/54	0.078^b^	0.962^b^
Grades (*n*)	83	Grade 0, 86	Grade I, 79; grade II, 12	NA	NA

*NA* not applicable

^a^ANOVA

^b^Chi-square test

### Levels of the four indicators

**Fig. 1:**
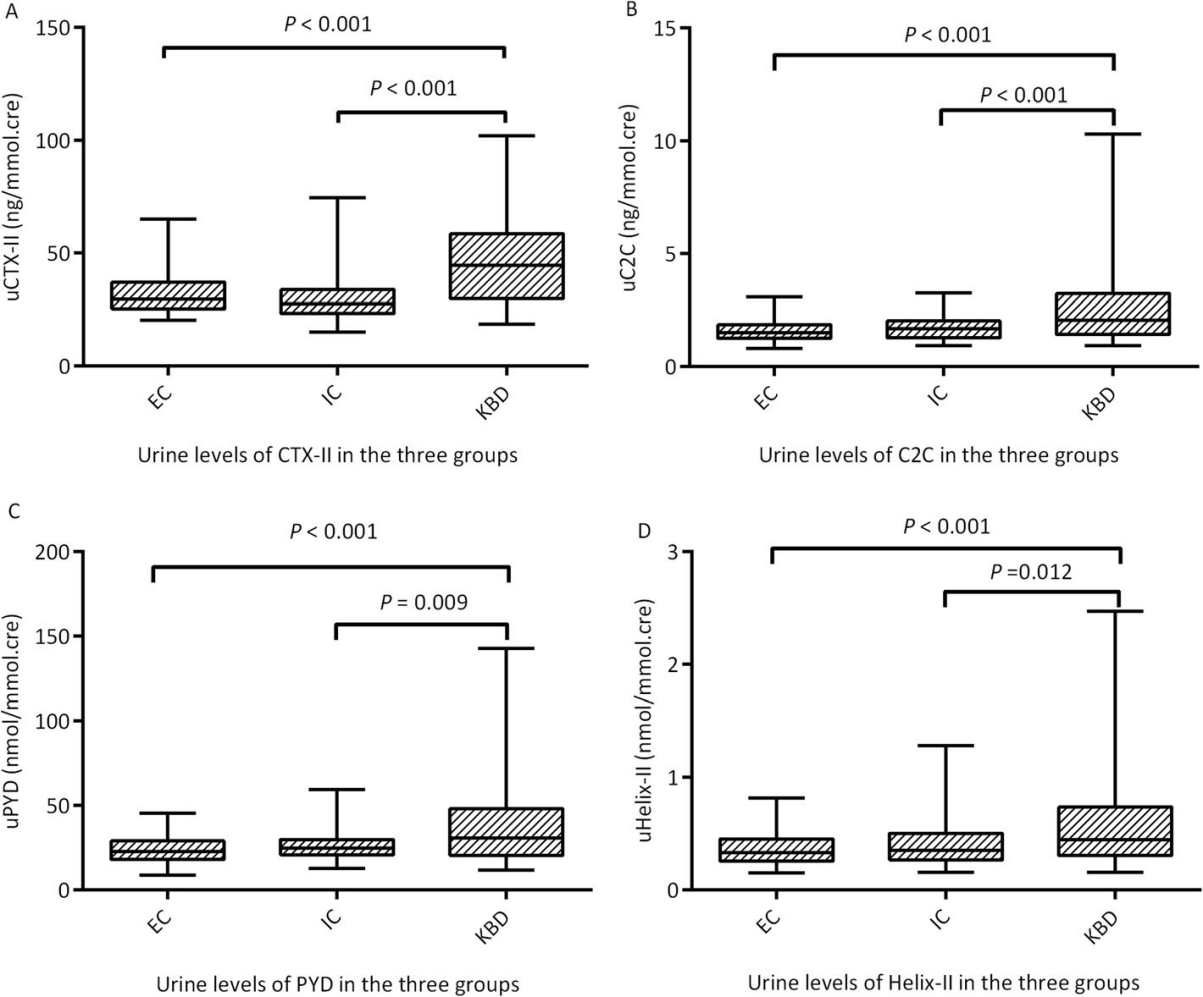
The levels of uCTX-II (**a**), uC2C (**b**), uPYD (**c**), and uHelix-II (**d**) in the EC group, the IC group, and the KBD group. The medians of the four biomarkers in the KBD group were significantly higher than those in the IC group (*H* = −6.512, *P* < 0.001; *H* = −3.813, *P* < 0.001; *H* = −2.973, *P* = 0.009; and *H* = −2.871, *P* = 0.012, respectively) and in the EC group (*H* = − 4.942, *P* < 0.001; *H* = − 4.837, *P* < 0.001; *H* = − 4.628, *P* < 0.001; and *H* = − 4.054, *P* < 0.001, respectively). There were no differences between the IC group and EC group (*H* = 1.490, *P* = 0.409; *H* = − 1.045, *P* = 0.889; *H* = − 1.659, *P* = 0.291; and *H* = − 1.194, *P* = 0.698, respectively)

The medians of uCTX-II, uC2C, uPYD, and uHelix-II in the EC group were 29.57 ng/mmol.cre, 1.49 ng/mmol.cre, 22.73 nmol/mmol.cre, and 0.33 nmol/mmol.cre, respectively, and they were 27.48 ng/mmol.cre, 1.67 ng/mmol.cre, 24.93 nmol/mmol.cre, and 0.35 nmol/mmol.cre in the IC group, respectively, while the medians in the KBD group were 44.39 ng/mmol.cre, 2.04 ng/mmol.cre, 30.93 nmol/mmol.cre, and 0.45 nmol/mmol.cre, respectively. In general, there were statistically significant differences among the three groups for each biomarker (*H* = 46.659, *P* < 0.001; *H* = 26.260, *P* < 0.001; *H* = 22.139, *P* < 0.001; *H* = 17.537, *P* < 0.001, for uCTX-II, uC2C, uPYD, and uHelix-II, respectively) (Table 2). Specifically, the medians of uCTX-II, uC2C, uPYD, and uHelix-II in the KBD group were significantly higher than those in the IC group (*H* = − 6.512, *P* < 0.001; *H* = − 3.813, *P* < 0.001; *H* = − 2.973, *P* = 0.009; and *H* = − 2.871, *P* = 0.012, respectively) and in the EC group (*H* = − 4.942, *P* < 0.001; *H* = − 4.837, *P* < 0.001; *H* = − 4.628, *P* < 0.001; and *H* = − 4.054, *P* < 0.001, respectively). There were no differences between the IC group and EC group (*H* = 1.490, *P* = 0.409; *H* = − 1.045, *P* = 0.889; *H* = − 1.659, *P* = 0.291; *H* = − 1.194, *P* = 0.698 for uCTX-II, uC2C, uPYD, and uHelix-II, respectively) (Fig. 1).

**Table 2:** Levels of uCTX-II, uC2C, uPYD, and uHelix-II in the EC group, IC group, and KBD group

Marker	EC		IC		KBD		*H*	*P*
	Median	*P*_25_, *P*_75_	Median	*P*_25_, *P*_75_	Median	*P*_25_, *P*_75_		
uCTX-II (ng/mmol.cre)	29.57	25.30, 37.05	27.48	23.20, 33.80	44.39	29.72, 58.58	46.659	< 0.001^c^
uC2C (ng/mmol.cre)	1.49	1.25, 1.86	1.67	1.27, 2.02	2.04	1.42, 3.23	26.260	< 0.001^c^
uPYD (nmol/mmol.cre)	22.73	18.13, 29.07	24.93	20.83, 29.88	30.93	20.67, 48.04	22.139	< 0.001^c^
uHelix-II (nmol/mmol.cre)	0.33	0.26, 0.45	0.35	0.27, 0.50	0.45	0.31, 0.74	17.537	< 0.001^c^

*EC* external control, *IC* internal control, *KBD* Kashin-Beck disease

^c^Kruskal-Wallis *H* test

### Correlation between sex and CTX-II, C2C, PYD, and Helix-II levels in urine

There were no significant differences in the expression of CTX-II, C2C, PYD, and Helix-II at different concentrations in the control group, the internal control group, or the KBD group (*P* > 0.05). Collectively, the median corrected concentrations of CTX-II, C2C, PYD, and Helix-II in male and female subjects in the external control group were 29.19 and 30.00 ng/mmol.cre (*Z* = − 0.102, *P =* 0.918), 1.49 and 1.51 ng/mmol.cre (*Z* = 1.051, *P =* 0.293), 21.23 and 23.39 nmol/mmol.cre (*Z* = 1.079, *P =* 0.280), and 0.35 and 0.33 nmol/mmol.cre (*Z* = 0.884, *P =* 0.377), respectively. In the internal control group, they were 30.28 and 26.65 ng/mmol.cre (*Z* = − 1.663, *P =* 0.096), 1.67 and 1.67 ng/mmol.cre (*Z* = − 0.718, *P =* 0.473), 24.98 and 24.93 nmol/mmol.cre (*Z* = − 0.455, *P =* 0.649), and 0.37 and 0.35 nmol/mmol.cre (*Z* = 0.420, *P =* 0.674), respectively, while those in the KBD group were 39.71 and 45.68 ng/mmol.cre (*Z* = − 0.040, *P =* 0.968), 1.99 and 2.06 ng/mmol.cre (*Z* = 0.283, *P =* 0.777), 30.44 and 32.07 nmol/mmol.cre (*Z* = 0.364, *P =* 0.716), and 0.45 and 0.46 nmol/mmol.cre (*Z* = 0.299, *P =* 0.765), respectively (Table 3).

**Table 3:** Urine levels of CTX-II, C2C, PYD, and Helix-II by sex among the external controls, internal controls, and KBD patients

Marker	Group	Male		Female		*Z*	*P*
		Median	*P*_25_, *P*_75_	Median	*P*_25_, *P*_75_		
CTX-II (ng/mmol.cre)	EC	29.19	25.17, 40.77	30	25.57, 35.00	− 0.102	0.918
	IC	30.28	24.75, 37.02	26.65	22.80, 32.34	− 1.663	0.096
	KBD	39.71	30.34, 62.45	45.68	29.02, 57.94	− 0.04	0.968
C2C (ng/mmol.cre)	EC	1.49	1.16, 1.73	1.51	1.27, 1.89	1.051	0.293
	IC	1.67	1.27, 2.17	1.67	1.25, 1.99	− 0.718	0.473
	KBD	1.99	1.35, 3.53	2.06	1.47, 3.08	0.283	0.777
PYD (nmol/mmol.cre)	EC	21.23	16.76, 28.50	23.39	18.93, 29.09	1.079	0.28
	IC	24.98	21.39, 30.72	24.93	19.72, 29.23	− 0.455	0.649
	KBD	30.44	20.77, 51.37	32.07	20.62, 46.49	0.364	0.716
Helix-II (nmol/mmol.cre)	EC	0.35	0.22, 0.45	0.33	0.28, 0.45	0.884	0.377
	IC	0.37	0.27, 0.48	0.35	0.27, 0.53	0.42	0.674
	KBD	0.45	0.31, 0.70	0.46	0.32, 0.80	0.299	0.765

### Usefulness of the four indicators for assisting in the diagnosis of KBD

**Fig. 2:**
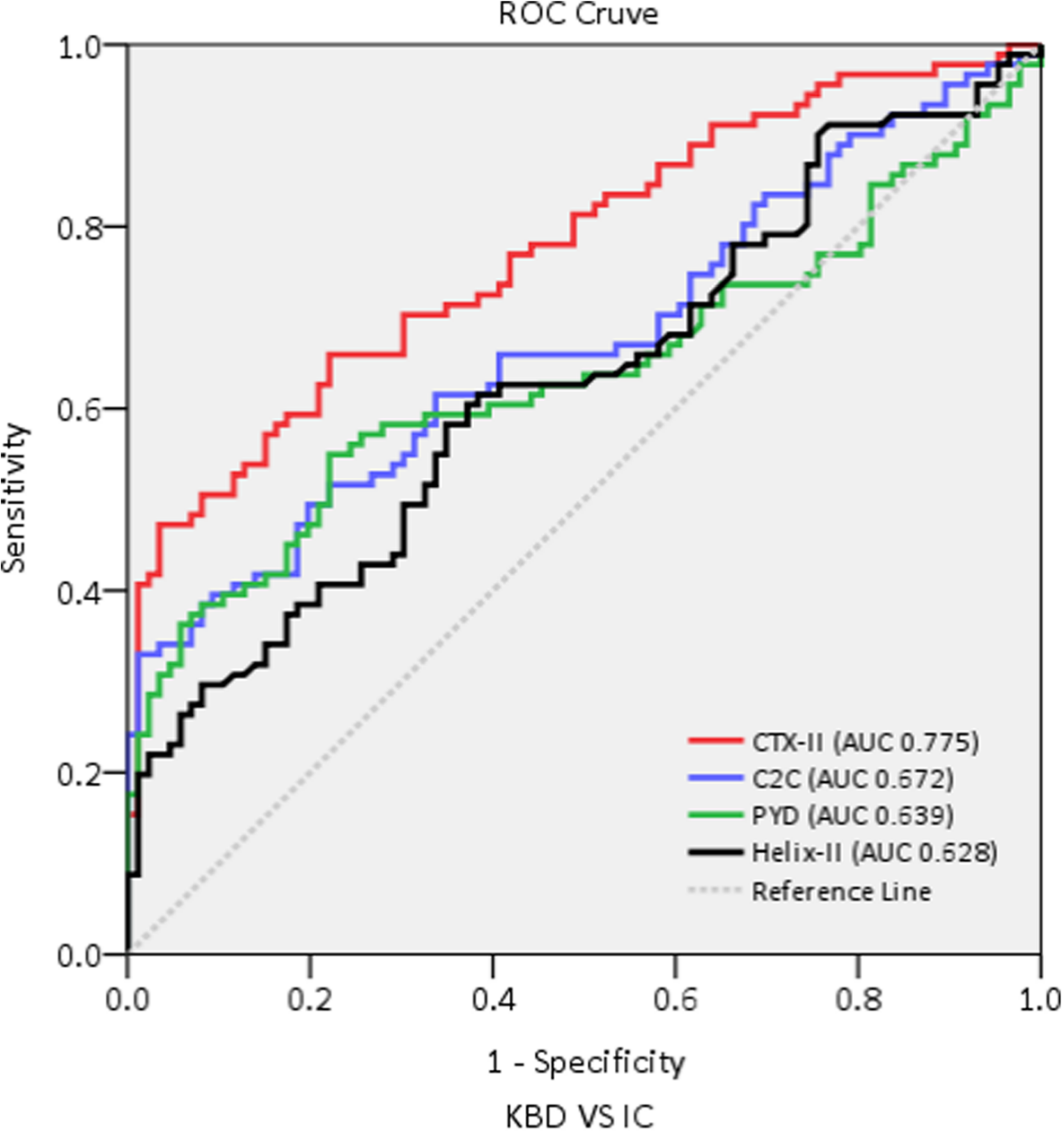
ROC curves of the KBD group versus the IC group. The AUC for uCTX-II, uC2C, uPYD, and uHelix-II was 0.775 (*P* < 0.001), 0.672 (*P* < 0.001), 0.639 (*P* = 0.001), and 0.628 (*P* = 0.003), respectively. The AUC of uCTX-II was higher than that of the others, and its diagnostic values were significantly higher than those of the other three indicators for KBD diagnosis

To investigate the diagnostic ability of each indicator to diagnose KBD, receiver operator characteristic (ROC) curves were utilized. The larger the area under the curve (AUC), the higher the diagnostic value of the marker. In the KBD diagnosis, the AUC for uCTX-II, uC2C, uPYD, and uHelix-II was 0.775 (*P* < 0.001), 0.672 (*P* < 0.001), 0.639 (*P* = 0.001), and 0.628 (*P* = 0.003), respectively. The AUC of uCTX-II was higher than that of the others, and its diagnostic values were significantly higher than those of the other three indicators for KBD diagnosis (Fig. 2).

### Relationship between the levels of the four indicators and KBD grades

In this study, 86.8% of KBD patients were grade I, and 13.2% were grade II. The median levels of the four markers for grade 0 KBD subjects (IC) were 27.48 ng/mmol.cre, 1.67 ng/mmol.cre, 24.93 nmol/mmol.cre, and 0.35 nmol/mmol.cre for uCTX-II, uC2C, uPYD, and uHelix-II, respectively. The median levels for grade I KBD patients were 40.15 ng/mmol.cre, 1.99 ng/mmol.cre, 30.54 nmol/mmol.cre, and 0.44 nmol/mmol.cre for uCTX-II, uC2C, uPYD, and uHelix-II, respectively, which were higher than those of grade 0 KBD patients. The medial levels for grade II KBD patients were 56.59 ng/mmol.cre, 3.01 ng/mmol.cre, 47.61 nmol/mmol.cre, and 0.67 nmol/mmol.cre for uCTX-II, uC2C, uPYD, and uHelix-II, respectively, which were higher than those of grade 0 and grade I KBD patients, as indicated on the box plots. In general, there were statistically significant differences among the three groups for each biomarker (*H* = 42.707, *P* < 0.001; *H* = 17.808, *P* < 0.001; *H* = 13.474, *P* = 0.001; *H* = 9.760, *P* = 0.008 for uCTX-II, uC2C, uPYD, and uHelix-II, respectively) (Table 4).

**Table 4:** Levels of uCTX-II, uC2C, uPYD, and uHelix-II in the different KBD grade groups

Markers	IC (grade 0 KBD)		Grade I KBD		Grade II KBD		*H*	*P*
	Median	*P*_25_, *P*_75_	Median	*P*_25_, *P*_75_	Median	*P*_25_, *P*_75_		
uCTX-II (ng/mmol.cre)	27.48	23.20, 33.80	40.15	29.63, 56.81	56.59	37.05, 77.76	42.707	< 0.001^c^
uC2C (ng/mmol.cre)	1.67	1.27, 2.02	1.99	1.39, 3.06	3.01	1.64, 4.45	17.808	< 0.001^c^
uPYD (nmol/mmol.cre)	24.93	20.83, 29.88	30.54	19.56, 43.51	47.61	28.14, 73.91	13.474	0.001^c^
uHelix-II (nmol/mmol.cre)	0.35	0.27, 0.50	0.44	0.31, 0.70	0.67	0.30, 1.29	9.760	0.008^c^

*IC* internal control, *KBD* Kashin-Beck disease

^c^Kruskal-Wallis *H* test

**Fig. 3:**
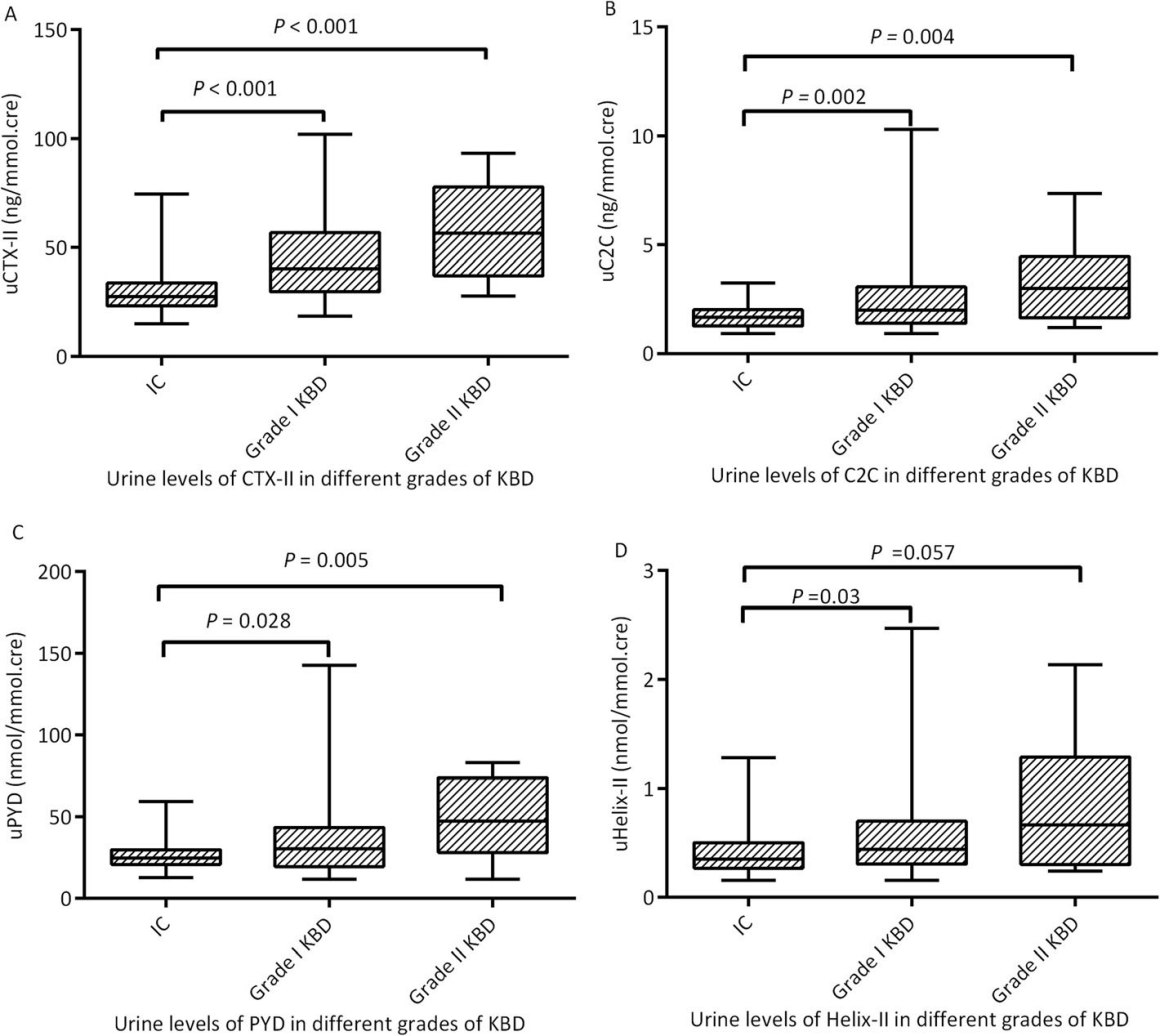
Levels of uCTX-II (**a**), uC2C (**b**), uPYD (**c**), and uHelix-II (**d**) in different KBD grades**.** The levels of the four markers in the grade I KBD group were significantly higher than those in the grade 0 KBD group (*H* = − 5.673, *P* < 0.001; *H* = − 3.419, *P* = 0.002; *H* = − 2.594, *P* = 0.028; and *H* = − 2.572, *P* = 0.030, respectively). The levels of uCTX-II (**a**), uC2C (**b**), and uPYD (**c**) in the grade II KBD group were significantly higher than those in the grade 0 KBD group (*H* = − 4.521, *P* < 0.001; *H* = − 3.228, *P* = 0.004; and *H* = − 3.148, *P* = 0.005, respectively). Although there were no statistically significant differences in the levels of uHelix-II (**d**) between the grade II KBD group and the grade 0 KBD group (*H* = − 2.344, *P* = 0.057, *P* value close to the significance level of 0.05), the uHelix-II level of the grade II KBD was significantly higher than that of the grade I KBD as seen in subpanel D. There were no statistically significant differences in the levels of the four markers between the grade II KBD group and the grade I KBD group (*H* = − 1.644, *P* = 0.301; *H* = − 1.491, *P* = 0.408; *H* = − 1.826, *P* = 0.204; and *H* = − 1.038, *P* = 0.898, respectively)

Specifically, the levels of uCTX-II (a), uC2C (b), uPYD (c), and uHelix-II (d) in the grade I KBD group were significantly higher than those in the grade 0 KBD group (*H* = − 5.673, *P* < 0.001; *H* = − 3.419, *P* = 0.002; *H* = − 2.594, *P* = 0.028; and *H* = − 2.572, *P* = 0.030, respectively). The levels of uCTX-II (a), uC2C (b), and uPYD (c) in the grade II KBD group were significantly higher than those in the grade 0 KBD group (*H* = − 4.521, *P* < 0.001; *H* = − 3.228, *P* = 0.004; and *H* = − 3.148, *P* = 0.005, respectively). Although there were no statistically significant differences in the levels of uHelix-II (d) between the grade II KBD group and the grade 0 KBD group (*H* = − 2.344, *P* = 0.057, *P* value close to the significance level of 0.05), the uHelix-II level of the grade II KBD was significantly higher than that of the grade I KBD patients as seen in subpanel d of Fig. 3. There were no statistically significant differences in the levels of uCTX-II (a), uC2C (b), uPYD (c), and uHelix-II (d) between the grade II KBD group and the grade I KBD group (*H* = − 1.644, *P* = 0.301; *H* = − 1.491, *P* = 0.408; *H* = − 1.826, *P* = 0.204; and *H* = − 1.038, *P* = 0.898, respectively) (Fig. 3).

## Discussion

KBD is a severe endemic osteoarthropathy that may lead to limb pain, joint deformation, cartilage destruction, inconvenient movement, physical disability, reduced labor ability, and difficulty with self-care. At present, the evaluation of osteochondritis (such as KBD and OA) mainly depends on X-rays or magnetic resonance imaging equipment. However, these methods are expensive, cumbersome, and even have a radiation hazard for the human body. Moreover, they are not sensitive to early pathological changes which occur before the onset of clinical signs of OA and KBD, and it is not possible to better evaluate early and subtle changes in bone and cartilage diseases [7]. Consequently, other ways to evaluate the cartilage changes associated with KBD need to be explored.

Urine sample collection is not traumatic for the human body. Meanwhile, molecular biomarkers in urine can provide direct information about metabolic changes in joint cartilage tissue, cartilage turnover, and local inflammation. The exploration of specific biomarkers in other types of osteoarthropathy (such as OA) that are linked with KBD provided some clues for uncovering a pathogenic mechanism for KBD in the present study. Here, we selected three groups of subjects. KBD patients and IC subjects were from KBD endemic areas, and EC subjects were from non-KBD endemic areas. The expression levels of the four biomarkers uCTX-II, uC2C, uPYD, and uHelix-II were detected. The levels of each marker in different groups were compared in detail. To select the best biomarker for KBD, the prediction accuracy of each marker was analyzed. Thereafter, the usefulness of the various markers for evaluating disease severity was explored. Basic differences in the expression levels of KBD in the different severity grades were compared.

CTX-II is a degradation of a 1/4 fragment of collagen II. Previous studies have shown that the uCTX-II level may reflect the extent of type II collagen degradation in patients with OA [8]. Recently, another study found that uCTX-II in patients with KBD was significantly increased [9]. In this study, the uCTX-II level in the KBD group was significantly higher than that in the IC group (confirming the conclusions of previous studies). The AUC of uCTX-II was higher than that of the other three markers in evaluating the diagnostic value of KBD. Therefore, uCTX-II may be a particularly valuable biomarker for diagnosing KBD and is a useful clue for future studies on KBD.

C2C is a degradation of a 3/4 fragment of collagen II. Previous studies have shown that the uC2C level may be associated with OA and may reflect the extent of cartilage degradation [10]. However, uC2C levels in KBD patients have not been previously reported. The uC2C level in the KBD group in this study was significantly higher than that in the control groups. It was discovered that there was also a positive correlation between the presence of KBD and the uC2C level, but uC2C was not better than uCTX-II for diagnosing KBD. The role that uC2C plays in KBD requires further study.

PYD (C_18_H_28_N_4_O_8_) is a derivative of a collagen linkage and a specific marker of bone and cartilage collagen; it is more sensitive than hydroxyproline and stabilizes the collagen chain [4]. The uPYD level is a specific and sensitive index reflecting bone resorption [11, 12]. Previous studies [13, 14] showed that there may be a correlation between uPYD and OA. uPYD may be a potential marker for assessing the severity of OA. Graverand [15] reported that uPYD levels were higher in patients with severe OA than in patients with mild OA. Increased PYD levels reflect bone erosion and/or sclerosing bone remodeling of osteophytes in the joints. In previous studies of KBD biomarkers, the uPYD level may also have reflected pathological bone metabolism in KBD patients. Dong [16] found that the average level of uPYD in the degree II group was higher than that in the degree I group and the control group. However, there was no significant difference between the degree I group and the control group. In addition, some studies have shown that estrogen has a major influence on uPYD levels. Consequently, pre-menopausal women were excluded in the experimental design as much as possible in this study. In the results, the level of uPYD in the KBD group was significantly higher than that in the IC group (consistent with previous studies). Therefore, the uPYD level is a diagnostic marker for KBD, but it is also not as good of a marker as uCTX-II.

Helix-II is a type II collagen degradation fragment with an epitope of ^642^ERGETGPOGTS^652^ (O is hydroxyproline) [5]. Previous studies [17, 18] suggested that the uHelix-II level may be associated with OA and that it could be used as a biomarker to reflect early cartilage damage but that it could not be used to evaluate the severity or progress of changes in knee cartilage injuries. However, studies of uHelix-II are rare in KBD. In this study, the uHelix-II level in the KBD group was significantly higher than that in the control groups; this is the first finding of this kind and indicates that there is also a correlation between the uHelix-II level and KBD. However, it was also not as good as uCTX-II for diagnosing KBD.

In the comparison of the levels of each indicator among the different KBD grades, although there were no significant differences between the levels of grade II KBD patients and grade I KBD patients, the median levels of the four markers in grade II KBD patients were significantly higher than those in grade 0 and I KBD patients, and the median levels in grade I KBD patients were significantly higher than those in grade 0 KBD patients. Therefore, there were some tendencies for these biomarker levels to increase with increasing KBD levels, demonstrating their usefulness for assessing the severity of KBD.

It is worth mentioning some highlights of our study. At first, this study initially found that the levels of C2C and Helix-II in the urine of adult patients with KBD were elevated, which suggested that the pathogenic process of KBD may also be related to the metabolism of the two biomarkers, and this needs further study.

In the course of the present study, the endemic characteristics of KBD were fully considered. At present, the pathogenesis of KBD is still not completely clear. Three main hypotheses have been developed: selenium deficiency, cereal contamination by mycotoxin-producing fungi, and high levels of organic material in drinking water. When exploring the pathogenic factors of KBD, the obvious regional features are very important clues and should be fully utilized. Due to the possible presence of environmental pathogenic factors in historic KBD epidemic areas, the control group was divided into an internal control group in KBD endemic areas and an external control group in non-KBD endemic areas, a design that was not found in previous studies of KBD. The results of this study showed that the levels of each marker were not significantly different between the internal control group and the external control group, suggesting that environmental pathogenic factors in KBD endemic areas may be well controlled or almost eliminated.

In addition, certain interference factors were excluded in advance of the present study. Previous studies [19, 20] suggested that estrogen may inhibit the degradation of the cartilage matrix to a certain extent. To minimize the interference of estrogen, the women recruited for data analysis were mostly elderly people who were postmenopausal.

In the process of finding the best diagnostic marker for KBD, uCTX-II was more powerful than the other three markers. This result provided an effective clue and reminded us that we should consider the factors leading to changes in the CTX-II level in urine, and this requires further exploration in the future.

Furthermore, in terms of practical value and cost savings, the urine sample may be the simplest, cheapest, and easiest alternative to mass population research of KBD in the future. At present, the gold standard for the diagnosis of bone and joint diseases is mainly X-rays and magnetic resonance imaging equipment, but they are costly, cumbersome to operate, harmful to the human body, and not sensitive to the early pathological changes that occur before the appearance of clinical symptoms of OA or KBD. Blood, synovial fluid, and other bodily fluids could be used to search for biomarkers of cartilage metabolism; however, these are difficult to collect, and the detection costs are high. In contrast, urine detection is random, noninvasive, easy to implement, and inexpensive. Therefore, we can preliminarily screen KBD patients with some urine-assisted diagnostic indicators and then confirm the diagnosis by X-ray. This can save a lot of manpower, material, and financial resources.

Nevertheless, there were some deficiencies in this study. The problems of small sample size and individual differences should not be ignored. The number of individuals in each group in the study was limited, and there were many individual differences in the subjects. Therefore, the conclusions of this study also need further verification.

## Conclusions

In summary, this study initially found that the levels of uC2C and uHelix-II in adult patients with KBD were elevated, which preliminarily revealed that the pathogenic process of KBD may be related to the metabolism of these two biomarkers. The levels of uCTX-II, uC2C, uPYD, and uHelix-II were increased in elderly KBD patients and showed an increasing trend as the severity of KBD increased. The results also showed that the prediction accuracy of uCTX-II was more useful than that of the other biomarkers for assisting in the diagnosis of KBD.

## Data Availability

The datasets used and/or analyzed during the current study are available from the corresponding author upon reasonable request.

## Abbreviations

AUC: Area under the curve
Cre: Creatinine
CTX-II: C II C-telopeptide
DPD: Deoxypyridinoline
EC: External control
ELISA: Enzyme-linked immunosorbent
Helix-II: Type II collagen helical peptide
IC: Internal control
KBD: Kashin-Beck disease
MMP: Matrix metalloproteinase
NTX-II: N-telopeptides of type II collagen
OA: Osteoarthritis
OC: Osteochondrosis
PYD: Pyridinoline
RA: Rheumatoid arthritis
ROC: Receiver operating characteristic
C2C: COL2-3/4C_long mono_

## References

[1] Stone R (2009). Diseases. A medical mystery in middle China. Science.

[2] Fosang AJ, Stanton H, Little CB (2003). Neoepitopes as biomarkers of cartilage catabolism. Inflamm Res.

[3] Poole AR, Ionescu M, Fitzcharles MA (2004). The assessment of cartilage degradation in vivo: development of an immunoassay for the measurement in body fluids of type II collagen cleaved by collagenases. J Immunol Methods.

[4] Uchiyama A, Inoue T, Fujimoto D (1981). Synthesis of pyridinoline during in vitro aging of bone collagen. J Biochem.

[5] Charni N, Juillet F, Garnero P (2005). Urinary type II collagen helical peptide (HELIX-II) as a new biochemical marker of cartilage degradation in patients with osteoarthritis and rheumatoid arthritis. Arthritis Rheum.

[6] National Health and Family Planning Commission of People’s Republic of China. Diagnosis of Kashin-Beck disease (WS/T 207–2010). 2010. (In Chinese).

[7] Roemer FW, Eckstein F, Hayashi D (2014). The role of imaging in osteoarthritis. Best Pract Res Clin Rheumatol.

[8] Kalai E, Bahlous A, Charni N (2012). Increased urinary type II collagen C-telopeptide levels in Tunisian patients with knee osteoarthritis. Clin Lab.

[9] Zhao ZJ, Pu GL, Zhan PZ (2017). Detection of the urinary biomarkers PYD, CTX-II, and DPD in patients with Kashin-Beck disease in the Qinghai province of China. Biomed Environ Sci.

[10] Jiang MY, Tang JC, Xin LY (2014). Detection of urine C2C level in patients with knee osteoarthritis. Chin J Osteopor.

[11] Halleen JM (2003). Tartrate-resistant acid phosphatase 5B is a specific and sensitive marker of bone resorption. Anticancer Res.

[12] Que WJ, Feng ZP. Research progress of biochemical markers in bone conversion. Chin J Osteopor. 2014(5):575–9. (In Chinese).

[13] Sarukawa J, Takahashi M, Doi M (2010). A longitudinal analysis of urinary biochemical markers and bone mineral density in STR/Ort mice as a model of spontaneous osteoarthritis. Arthritis Rheum.

[14] Tanimoto K, Ohno S, Imada M (2004). Utility of urinary pyridinoline and deoxypyridinoline ratio for diagnosis of osteoarthritis at temporomandibular joint. J Oral Pathol Med.

[15] Graverand MP, Tron AM, Ichou M (1996). Assessment of urinary hydroxypyridinium cross-links measurement in osteoarthritis. Br J Rheumatol.

[16] Dong W, Zhang Y, Liu H (2009). Detection of unsaturated disaccharides, pyridinoline, and hydroxyproline in urine of patients with Kashin-Beck disease: comparison with controls in an endemic area. J Rheumatol.

[17] Garnero P, Charni N, Juillet F (2006). Increased urinary type II collagen helical and C-telopeptide levels are independently associated with a rapidly destructive hip osteoarthritis. Ann Rheum Dis.

[18] Wei X, Yin K, Li P (2013). Type II collagen fragment HELIX-II is a marker for early cartilage lesions but does not predict the progression of cartilage destruction in human knee joint synovial fluid. Rheumatol Int.

[19] Bay-Jensen AC, Tabassi NC, Sondergaard LV (2009). The response to oestrogen deprivation of the cartilage collagen degradation marker, CTX-II, is unique compared with other markers of collagen turnover. Arthritis Res Ther.

[20] Christgau S, Tanko LB, Cloos PA (2004). Suppression of elevated cartilage turnover in postmenopausal women and in ovariectomized rats by estrogen and a selective estrogen-receptor modulator (SERM). Menopause.

